# Two New Sphingolipids from the Leaves of *Piper betle* L.

**DOI:** 10.3390/molecules180911241

**Published:** 2013-09-12

**Authors:** Duo-Zhi Chen, Hua-Bin Xiong, Kai Tian, Jun-Ming Guo, Xiang-Zhong Huang, Zhi-Yong Jiang

**Affiliations:** Key Laboratory of Chemistry in Ethnic Medicinal Resources, State Ethnic Affairs Commission & Ministry of Education, Yunnan University of Nationalities, Kunming 650500, Yunnan, China

**Keywords:** *Piper betle* L., Piperaceae, sphingolipids, spectroscopic analysis, cytotoxic

## Abstract

Two new sphingolipids, pipercerebrosides A (**1**) and B (**2**), were isolated from the leaves of *Piper betle* L. Their structures, including absolute configurations, were determined by spectroscopic analysis and chemical degradation. These two compounds did not show significant cytotoxic activity against the cancer cell lines K562 and HL-60 in a MTT assay.

## 1. Introduction

The stems and/or leaves of *Piper betle* L., an evergreen perennial creeper plant widely distributed in the south of China, are used as Chinese Traditional Medicines to cure stomachache, wind-cold cough, sore and furuncle, and eczema [1]. This plant has been known to contain chemical compounds, such as alkaloids, steroids, phenols, and terpenes, with a wide range of bioactivities, like antioxidant, antifungal, antiulcerogenic, antiplatelet, antidiabetic, anti-inflammatory, antifilarial, and antimicrobial activity [2,3,4].

In a previous study, we extracted *P. betle* stems collected from western China with acetone. This extract, from which eight piper amides and two lignans were isolated, showed antioxidant activity in a 2,2-diphenyl-1-picrylhydrazyl radical (DPPH), scavenging assay and antibacterial activity in a disc diffusion test [5]. Our continuing studies on the leaves of the plant has now led to the isolation of two new sphingolipids, pipercerebrosides A (**1**) and B (**2**) and other five known sphingolipids, 3-octadecenamide, 3-eicosenamide, agelasphin-11, agelasphin-7a and agelasphin-13. Sphingolipids have been found to have anti-tumor, immunostimulatory, neuritogenic, antiviral, antifungal, and nematicidal activities [6,7,8]. In this paper we present the isolation and structural elucidation of the new compounds.

## 2. Results and Discussion

### 2.1. Structure Analysis of Pipercerebroside A

Compound **1** was isolated as a white amorphous powder. Its molecular formula was determined to be C_35_H_67_NO_10_ by HRESIMS (*m/z* 662.4797 [M + H]^+^, calcd. for 662.4799). The IR spectrum of **1** showed absorption bands for hydroxyl groups at 3,385 cm^−1^ and for a secondary amide at 1,653 cm^−1^. The ^1^H- and ^13^C-NMR (Table 1) spectra of **1** indicated the presence of a β-d-glucopyranosyl moiety (*δ*_H_ 4.14, 1H, d, *J* = 7.6 Hz, anomeric proton; *δ*_C_ 103.9, 73.9, 77.3, 70.4, 76.9, and 61.5), an amide linkage (*δ*_H_ 7.55, 1H, d, *J* = 8.6 Hz; *δ*_C_ 174.3), two olefinic methines [*δ*_H_ 5.28 (1H, dt, *J* = 10.0, 5.0 Hz) and 5.35 (1H, dt, *J* = 10.0, 5.0 Hz); *δ*_C_ 129.5 and 130.3], an amidomethine (*δ*_H_ 3.97, *δ*_C_ 50.4), an oxygenated methylene (*δ*_H_ 3.96 and 3.66; *δ*_C_ 69.5), three oxygenated methines (*δ*_H_ 3.46, 3.53 and 3.81; *δ*_C_ 71.1, 74.3 and 71.5), two terminal methyls (*δ*_H_ 0.83, 6H, t, *J* = 6.5 Hz) and two long-chain aliphatic moieties appearing as a multiplets (*δ*_H_ 1.20-1.30). All of the above spectral data revealed that **1** was a glycosphingolipid [9,10]. 

**Table 1:** ^1^H (500 MHz) and ^13^C-NMR (125 MHz) data for **1** (in DMSO) and **2** (in pyridine-*d*_5_) *^a^*.

No.	1			No.	2	
	*δ*_H_	*δ*_C_			*δ*_H_	*δ*_C_
1a	3.66 (dd, 1H, 10.5, 4.0 Hz)3.96 (dd, 1H, 10.5, 4.0 Hz)	69.5 t		1a	4.42 (dd, 1H, 10.0, 4.5 Hz)4.50 (dd, 1H, 10.0, 4.5 Hz)	62.9 t
1b				1b		
2	3.97 (m, 1H)	50.4 d		2	5.16 (m, 1H)	54.0 d
3	3.53 (m, 1H)	74.3 d		3	4.35 (m, 1H)	77.1 d
4	3.46 (m, 1H)	71.1 d		4	4.30 (m, 1H)	73.9 d
5a	1.48 (m, 1H)	32.1 t		5a	2.02 (m, 1H)	34.2 t
5b	1.90 (m, 1H)			5b	2.19 (m, 1H)	
6	1.20–1.30 (m, 2H)	26.1 t		6	1.96 (m, 2H)	27.5 t
7	1.97 (m, 2H)	27.4 t		7–9	1.26–1.32 (m, 6H)	30.5–30.9 t
8	5.35 (dt, 1H, 10.0,5.0 Hz)	130.3 d		10a	2.05 (m, 1H)	34.2 t
9	5.28 (dt, 1H, 10.0,5.0 Hz)	129.5 d		10b	2.29 (m, 1H)	
10	1.97 (m, 2H)	27.2 t		11	5.52 (dt, 1H, 15.0, 6.0 Hz)	131.7
11–14	1.20–1.30 (m, 8H)	29.0–29.6t		12	5.52 (dt, 1H, 15.0, 6.0 Hz)	131.6
15	1.20–1.30 (m, 2H)	31.8 t		13a	2.03 (m, 1H)	33.8 t
16	1.20–1.30 (m, 2H)	22.6 t		13b	2.17 (m, 1H)	
17	0.83 (t, 3H, 6.5 Hz)	14.2 q		14–20	1.26–1.32 (m, 14H)	30.5–30.9 t
NH	7.55 (d, 1H, 8.6 Hz)			21	1.26–1.32 (m, 2H)	33.0 t
1′		174.3 s		22	1.26–1.32 (m, 2H)	23.8 t
2′	3.81 (dd, 1H, 7.5, 4.0 Hz)	71.5 d		23	0.87 (t, 3H, 6.5 Hz)	15.1 q
3′a	1.48 (m, 1H)	34.8 t		NH	8.58 (d, 1H, 9.0 Hz)	
3′b	2.00 (m, 1H)			1′		174.9 s
4′	1.20–1.30 (m, 2H)	24.9 t		2′	4.56 (dd, 1H, 7.5, 4.0 Hz)	73.7 d
5′–9′	1.20–1.30 (m, 10H)	29.0–29.6t		3′a	2.05 (m, 1H)	34.8 t
10′	1.20–1.30 (m, 2H)	31.8 t		3′b	2.21 (m, 1H)	
11′	1.20–1.30 (m, 2H)	22.6 t		4′a	1.65 (m, 1H)	
12′	0.83 (t, 3H, 6.5 Hz)	14.2 q		4′b	1.96 (m, 1H)	27.4 t
1′′	4.14 (d, 1H, 7.6 Hz),	103.9		5′–21′	1.26–1.32 (m, 32H)	30.5–30.9 t
2′′	2.95 (t, 1H, 8.0 Hz)	73.9		22′	1.26–1.32 (m, 2H)	33.0 t
3′′	3.13 (m, 1H)	77.3		23′	1.26–1.32 (m, 2H)	23.8 t
4′′	3.02 (m, 1H)	70.4		24′	0.87 (t, 3H, 6.5 Hz)	15.1 q
5′′	3.10 (m, 1H)	76.9				
6′′a	3.56 (dd, 1H, 12.0,5.0Hz)	61.5				
6′′b	3.70 (m, 1H)					

*^a^* Signals were assigned by means of ^1^H-^1^H COSY, HSQC and HMBC.

**Scheme 1:**
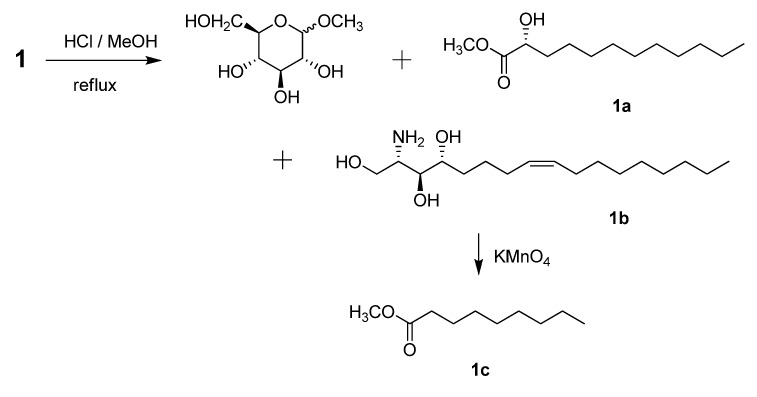
Methanolysis, oxidation and methylation of pipercerebroside A.

Analysis of the ^1^H-^1^H COSY, HMQC, and HMBC spectra led to the assignment of proton and carbon signals for **1**. Methanolysis of **1** yielded a fatty acid methyl ester **1a** and a long-chain base **1b** (Scheme 1). Compound **1a** was identified as 2-hydroxydodecanoic acid methyl ester 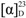 −1.2 (*c* 0.07, CHCl_3_) by means of GC/MS analysis, and the absolute configuration of C-2′ was determined to be *R* from the specific rotation [11]. The phytosphingosine part is thus a C_17_ aliphatic amino alcohol unit with three hydroxyls, an amino group, and an olefinic bond. The 2*S*, 3*S*, and 4*R* configurations of the ceramide moieties were assigned by comparing the specific rotation 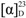 +9.6 (*c* 0.11, pyridine)], ^1^H-NMR, and ^13^C-NMR data of compound **1** with those of the known synthetic ceramide (2*S*,3*S*,4*R*)-2-[(2′*R*)-2′-hydroxytetracosanoylamino]-1,3,4-hexadecanetriol [12] and the natural ceramide 1-*O*-β-d-glucopyranosyl-(2*S*,3*S*,4*R*,8*E*)-2-[(2′*R*)-2′-hydroxybehenoylamino]-8-octadecene-1,3,4-triol [13]. To determine the position of the olefinic bond in the dihydrosphingosine moiety, KMnO_4_ oxidation [14] was performed on compound **1b** to yield nonanoic acid (**1a**) (Scheme 1), which was methylated and detected by GC/MS. This indicated the location of the olefinic bond between C-8 and C-9. The ∆ [8,9] olefinic bond was confirmed to have a (*Z*)-configuration as evidenced by the vicinal coupling constants (*J* = 10.0 Hz), together with the chemical shifts of C-7 (*δ* 27.4) and C-10 (*δ* 27.2). Analysis of the ^1^H-^1^H COSY, HMQC, and HMBC spectra led to the assignment of proton and carbon signals for **1**. Methanolysis of **1** yielded a fatty acid methyl ester **1a** and a long-chain base **1b** (Scheme 1). Compound **1a** was identified as 2-hydroxydodecanoic acid methyl ester 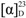 −1.2 (*c* 0.07, CHCl_3_) by means of GC/MS analysis, and the absolute configuration of C-2′ was determined to be *R* from the specific rotation [11]. The phytosphingosine part is thus a C_17_ aliphatic amino alcohol unit with three hydroxyls, an amino group, and an olefinic bond. The 2*S*, 3*S*, and 4*R* configurations of the ceramide moieties were assigned by comparing the specific rotation 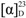 +9.6 (*c* 0.11, pyridine)], ^1^H-NMR, and ^13^C-NMR data of compound **1** with those of the known synthetic ceramide (2*S*,3*S*,4*R*)-2-[(2′*R*)-2′-hydroxytetracosanoylamino]-1,3,4-hexadecanetriol [12] and the natural ceramide 1-*O*-β-d-glucopyranosyl-(2*S*,3*S*,4*R*,8*E*)-2-[(2′*R*)-2′-hydroxybehenoylamino]-8-octadecene-1,3,4-triol [13]. To determine the position of the olefinic bond in the dihydrosphingosine moiety, KMnO_4_ oxidation [14] was performed on compound **1b** to yield nonanoic acid (**1a**) (Scheme 1), which was methylated and detected by GC/MS. This indicated the location of the olefinic bond between C-8 and C-9. The ∆ [8,9] olefinic bond was confirmed to have a (*Z*)-configuration as evidenced by the vicinal coupling constants (*J* = 10.0 Hz), together with the chemical shifts of C-7 (*δ* 27.4) and C-10 (*δ* 27.2) .

**Figure 1:**
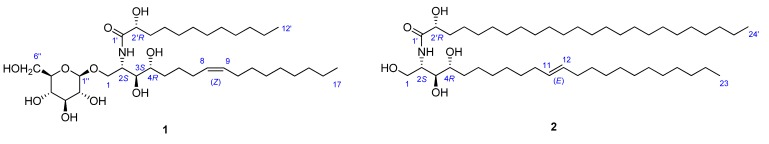
Structures of two new sfingolipids (pipercerebrosides) from the leaves of *Piper betle* L.

Due to the fact that signals of the carbons adjacent to a (*Z*)-configuration olefinic bond usually appear at *δ* 27.0–28.0 [15], whereas those of a (*E*)-configuration olefinic bond normally appear at *δ* 32.0–34.0 [9,16]. Consequently, the structure of **1** was determined to be 1-*O*-(*β*-D-glucopyranosyl)-(2*S*,3*S*,4*R*,8*Z*)-2-{[(2*R*)-2-hydroxyldodecanoyl] amino}heptadec-8-ene-1,3,4-triol (Figure 1), named pipercerebroside A.

### 2.2. Structure Analysis of Pipercerebroside B

Pipercerebroside B was isolated as a white amorphous powder. Its molecular formula was determined to be C_46_H_91_NO_5_ by HRESIMS (*m/z* 738.6966 [M + H]^+^, calcd. for 738.6975). The IR spectrum of **2** showed absorption bands for hydroxyl groups at 3,480 cm^−1^ and for a secondary amide at 1,640 cm^−1^. The ^1^H- and ^13^C-NMR (Table 1) spectra of **2** indicated the presence of an amide linkage (*δ*_H_ 8.58, 1H, d, *J* = 9.0 Hz; *δ*_C_ 174.9), two olefinic methines [*δ*_H_ 5.52 (2H, dt, *J* = 15.0, 6.0 Hz); *δ*_C_ 131.6 and 131.7], an amidomethine (*δ*_H_ 5.16, *δ*_C_ 54.0), an oxygenated methylene (*δ*_H_ 4.50 and 4.42; *δ*_C_ 62.9), three oxygenated methines (*δ*_H_ 4.30, 4.35 and 4.56; *δ*_C_ 73.9, 77.1 and 73.7), and Two terminal methyls (*δ*_H_ 0.87, 6H, t, *J* = 6.5 Hz) and two long-chain aliphatic moieties appearing as a multiplets (*δ*_H_ 1.26–1.32). All of the above spectral data revealed that **2** was also a sphingolipid [17,18]. Analysis of the ^1^H-^1^H COSY, HMQC, and HMBC spectra assigned the proton and carbon signals for **2**. Methanolysis of **2** yielded a fatty acid methyl ester (FAME) and a long chain base (LCB). The FAME compound was identified as 2-hydroxydodecanoic acid methyl ester by means of GC/MS analysis {EI: *m/z* 398, [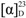 −1.4 (*c* 0.09, CHCl_3_)]} and the absolute configuration of C-2′ was determined to be *R* as in compound **1**. Thus, the LCB part is a C_23_ aliphatic amino alcohol unit containing three hydroxyls, an amino group and a double bond. The dihydrosphingosine (LCB) moiety was oxidized to yield undecanoic acid, which was methylated and detected by GC/MS. This indicated that the double bond was located at C-11. The 11,12 alkene bond was shown to be *trans* by the large vicinal coupling constants (*J* = 15.0). The *trans* geometry of this double bond was also supported by the chemical shift of C-10 (*δ* 34.2) and C-13 (*δ* 33.8).

Usually, the signals of the carbons adjacent to a *trans* double bond appear at *δ* 32-34, while those of a *cis* double bond appear at *δ* 27–28 [9,16]. Therefore, the structure of **2** was determined to be (2*S*,3*S*,4*R*,11*E*)-2-{[(2*R*)-2-hydroxytetracosanoyl] amino}tricos-11-ene-1,3,4-triol (Figure 1), named pipercerebroside B.

### 2.3. Cytotoxic Activity of Compounds

The two compounds were evaluated for their cytotoxic activity. None of them showed considerable inhibitory cytotoxic activity against cancer cell lines K562 and HL-60 at the concentration of 10 μM in a MTT assay.

## 3. Experimental

### 3.1. General

All reagents were analytical grade and water was distilled twice. TLC was preformed with silica gel GF254 (Marine Chemical Industry Factory, Qingdao, China), and the spots were visualized by spraying with 10% H2SO4/EtOH reagent. Column chromatography was performed using silica gel (Marine Chemical Industry Factory, Qingdao, China), reverse-phase C18 silica gel (Merck, Darmstadt, Germany) and Sephadex LH-20 (Sigma). Melting points were measured with an X-4 melting point apparatus and are uncorrected. Optical rotations were recorded on a Perkin-Elmer 241 polarimeter. UV spectra were obtained on a Perkin-Elmer Lambda 900 UV/VIS/NIR spectrophotometer. IR spectra were obtained on a Perkin-Elmer 577 spectrometer with KBr pellets.-NMR Spectra were recorded on a Bruker AV 500 spectrometer (^1^H, 500 MHz; ^13^C, 125 MHz), and chemical shifts are presented as values relative to tetramethylsilane as an internal standard. Low-resolution electrospray-ionization mass spectrometry (ESI-MS) and HR-ESI-MS were recorded on a Finnigan LCQ-Advantage mass spectrometer and a VG Auto-Spec-3000 mass spectrometer. Gas chromatography-mass spectrometer (GC-MS) experiments were performed on a Shimadzu GC-17A gas chromatograph apparatus (DB-5 capillary column: 30 m × 0.25 mm × 0.25 μm, helium flow rate: 0.8 ml/min) attached to a Shimadzu GCMS-QP5050A mass spectrometry equipped with an electron impact (EI) ion source (70 eV). The column was maintained at 100 °C for 1 min and then ramped from 100 to 260 °C at a rate of 4 °C/min with a final hold at 260 °C for 15 min.

### 3.2. Plant Resource

The leaves of *Piper betle* were collected from Baoshan city of Yunnan Province in China, in October 2010. It was identified by Prof. Shaobin Ma (Department of Biology, Yunnan University). The stems were harvested and air-dried at room temperature in shade. A voucher specimen (No. 20101020) was deposited in School of Chemistry and Biotechnology, Yunnan University of Nationalities, China.

### 3.3. Extraction and Isolation of New Compounds

The dried leaves (6.8 kg) of *P*. *betle* were extracted with 70% acetone (40 L × 3) under reflux (4 h). The acetone extract was evaporated to almost dryness *in vacuo*, and the resulting mixture (760 g) was suspended in water before being successively partitioned with petroleum ether, ethyl acetate, and *n*-butanol (2 L × 7). The ethyl acetate phase was concentrated to produce a black mass (177 g), which was separated into 11 fractions on a silica gel column using step gradient elution with CHCl_3_-MeOH (1:0–0:1). Fraction 3 (5.2 g) was applied to a Sephadex LH-20 column using MeOH-CHCl_3_ (1:2) as a solvent to afford seven subfractions. Subfraction 3–5 (2.7 g) was similarly chromatographed on a Sephadex LH-20 column eluted from 50% MeOH in CHCl_3_ to obtain eight fractions, of which the second fraction (230 mg) was purified by an RP-18 silica gel column eluted with MeOH-H_2_O (70:30–85:15) to provide compound **1** (28 mg). Subfraction 3–2 (640 mg) was subjected to a silica gel column using CHCl_3_-MeOH (20:1) as the eluent, yielding compound **2** (13 mg).

### 3.4. Methanolysis, Oxidation and Methylation of Pipercerebroside A

Pipercerebroside A (3 mg) was refluxed with 0.9 mol L^−1^ HCl in 82% aqueous MeOH (5 mL) for 18 h [19]. The resulting solution was extracted three times with *n*-hexane. The *n*-hexane solution was washed with water (5 mL) and dried over anhydrous Na_2_SO_4_ then concentrated to yield compound **1a** (1.1 mg). Compound **1a** was identified by analysis of GC-MS. After evaporation of MeOH, the H_2_O layer was neutralized with ammonia liquid and extracted with Et_2_O. The Et_2_O layer was then dried over anhydrous Na_2_SO_4_ and concentrated to obtain compound **1b** (0.8 mg). *2-Hydroxy-dodecanoic acid methyl ester* (**1a**): colorless oil, 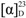 -1.2 (*c* 0.07, CHCl_3_); GC-MS: GC, *t*_R_ 39.41 min, EI-MS *m/z*: 171 [M − CH_3_OCO]^+^ (39), 127 [C_9_H_19_]^+^ (6), 111 (17), 97 (74), 90 [CH_3_OC(OH)=CH OH]^+^ (45), 83 (72), 69 (89), 55 (91), 43 (100).

Compound **1b** (0.8 mg) was dissolved in 10% H_2_SO_4_ and acetone (2.0 mL each). KMnO_4_ (50 mg) was then added to the solution and stirred overnight at room temperature [16,20]. The reaction was then quenched with aqueous Na_2_S_2_O_3_ (5%). After removal of acetone, the reaction mixture was extracted with Et_2_O. The Et_2_O layer was dried over Na_2_SO_4_ and concentrated to yield the residue, which was methylated using CH_2_N_2_ in Et_2_O at 0 °C [20]. The reaction mixture was kept in ice for 30 min before being allowed to stand at room temperature overnight. This procedure resulted in compound **1c** (0.4 mg), which was identified as methyl nonanoate by GC-MS. GC, *t*_R_ 21.30 min, EI-MS *m/z*: 172 [M]^+^ (5), 141 [M − OCH_3_]^+^ (11), 129 (9), 87 (46), 74 (100), 69 (7), 55 (19), 43 (23).

### 3.5. Methanolysis, Oxidation and Methylation of Pipercerebroside B

Pipercerebroside B (3 mg) was treated under the same conditions mentioned above to afford 2-hydroxytetracosanoic acid methyl ester (0.9 mg) and methyl undecanoate (0.3 mg). *2-Hydroxytetracosanoic acid methyl ester*: colorless oil, 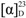 -1.4 (*c* 0.09, CHCl_3_); GC-MS: GC, *t*_R_ 54.38 min, EI-MS *m/z*: 398 [M]^+^ (11), 339 [M − CH_3_OCO]^+^ (9), 281 (5), 207 (10), 145 (4), 127 (6), 111 (12), 97 (27), 90 [CH_3_OC(OH)=CHOH]^+^ (28), 83 (27), 57 (58), 44 (100). *Methyl undecanoate*: GC-MS: GC, *t*_R_ 24.36 min, EI-MS *m/z*: 200 [M]^+^ (4), 169 [M − OCH_3_]^+^ (5), 157 (4), 143 (7), 101 (6), 87 (47), 74 (100), 69 (8), 55 (16), 43 (22).

### 3.6. Spectral data of New Compounds

*Pipercerebroside A*: white amorphous powder; mp 191–192 °C; 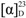 + 9.6 (*c* 0.11, pyridine); IR (KBr) *ν*_max_: 3385, 3178, 2922, 2853, 1653, 1543, 1463, 1402, 1083, 932 cm^−1^; ESI(+)-MS *m/z*: 662 [M + H]^+^, 684 [M + Na]^+^, 500 [M + H − glu]^+^; HRESI(+)-MS *m/z*: 662.4797 [M + H]^+^ (calcd. for C_35_H_68_O_10_N, 662.4799).

*Pipercerebroside B*: white amorphous powder; mp 191–192 °C; 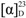 + 3.7 (*c* 0.19, pyridine); IR (CH_3_OH) *ν*_max_: 3480, 2940, 2860, 1640, 1506, and 1297 cm^−1^; ESI(+)-MS *m/z*: 738 [M + H]^+^; HRESI(+)-MS *m/z*: 738.6966 [M + H]^+^ (calcd. for C_46_H_92_NO_5_, 738.6975).

### 3.7. Bioassay

Inhibition of cell-growth activity was determined by a MTT assay using human chronic myelogenous leukemia cells (K562) and human promyelocytic leukemia cells [21]. *cis*-Diamminedichloroplatinum (DDP) was used as a positive control. None of the compounds showed any obvious cytotoxic effect against the cancer cell lines K562 and HL-60.

## 4. Conclusions

Two new sphingolipids, pipercerebrosides A (**1**) and B (**2**), were isolated from the leaves of *Piper betle.* L. Their structures including absolute configurations were determined by spectroscopic analysis 1D-NMR, 2D-NMR and MS experiment and chemical degradation. None of the compounds showed any significant cytotoxic activity against the cancer cell lines K562 and HL-60 at a concentration of 10 μM in the MTT assay.
